# Dosing Colistimethate Every 8 h Results in Higher Plasma Concentrations of Active Colistin Than Every 12-Hourly Dosing without Increase in Nephrotoxicity: A Phase 1 Pharmacokinetics Trial in Healthy Adult Volunteers

**DOI:** 10.3390/antibiotics11040490

**Published:** 2022-04-06

**Authors:** George A. Yendewa, John McLeod Griffiss, Wesley A. Gray, Amanda Healen, Howard M. Proskin, Scott A. Fulton, Mary Ann O’Riordan, Charles Hoppel, Jeffrey L. Blumer, Robert A. Salata

**Affiliations:** 1 Department of Medicine, Case Western Reserve University, Cleveland, OH 44106, USA; amb233@case.edu (A.H.); scott.fulton@uhhospitals.org (S.A.F.); robert.salata@uhhospitals.org (R.A.S.); 2 Division of Infectious Diseases and HIV Medicine, University Hospitals Cleveland Medical Center, Cleveland, OH 44106, USA; 3 ClinicalRM, Hinckley, OH 44233, USA; crapaud@loursage.org; 4 Smithers Rapra, Akron, OH 44303, USA; wgrayxx0@gmail.com; 5 Howard M. Proskin & Associates, Inc., Rochester, NY 14618, USA; hproskin@hmproskin.com; 6 Department of Pediatrics, Case Western Reserve University, Cleveland, OH 44106, USA; mao@case.edu; 7 Department of Pharmacology, Case Western Reserve University, Cleveland, OH 44106, USA; clh5@po.cwru.edu; 8 Department of Pediatrics, University of Toledo, Toledo, OH 43606, USA; jxb53@case.edu

**Keywords:** colistin, pharmacokinetics, gram-negative bacteria, antimicrobial resistance

## Abstract

Despite its use for decades, pharmacokinetic (PK) and safety studies on colistin are limited. We conducted a phase l, open-label trial to evaluate the safety and PK of multiple doses of intravenous (IV) and aerosolized colistimethate sodium (CMS) administered separately and in combination. In total, 31 healthy adults were enrolled into three cohorts of 9, 10, and 12 participants, respectively. Each cohort received increasing doses of CMS over three dosing periods as follows: Period 1 (IV only), 2.5 mg/kg every 12 h (q12h) to 3.3 mg/kg every 8 h (q8h); Period 2 (aerosolized only), 75 mg 2–4 doses, and Period 3 (combined IV aerosolized), in which was Periods 1 and 2 combined. Safety assessments, serum and lung concentrations of colistin analytes (colistin A, colistin B, CMS A, and CMS B), and kidney biomarkers were measured at specified time points. Increasing the CMS dose from 2.5 mg/kg q12h to q8h resulted in a 33% increase in serum colistin A concentrations from 3.9 μg/mL to 5.3 μg/mL—well above the accepted target of 2 μg/mL for 6 h after dosing, without evidence of nephrotoxicity. However, there was an increase in neurotoxicity, primarily perioral and lingual paresthesias, and self-limited ataxia. IV administration did not increase the lung concentrations of colistin.

## 1. Introduction

Colistin (also known as polymyxin E) is a decades-old antibiotic that has re-emerged to become a “last resort” regimen used in the treatment of infections caused by multidrug-resistant (Gram-negative) organisms (MDROs). It is a complex mixture of over 30 different polymyxins, with the two major components, colistin A and colistin B, accounting for over 85% of the total mass [1]. It is dosed as an inactive prodrug, colistimethate sodium (CMS), which is converted to active colistin via non-enzymatic hydrolysis in vivo [2]. Colistin is administered intramuscularly (IM), intravenously (IV), or via inhalation [3]. It is avidly reabsorbed in the kidney [3]; CMS, on the other hand, is excreted mostly through the kidneys and is associated with nephrotoxicity and neurotoxicity [3,4].

Discovered in Japan in 1949 and adopted throughout Europe in the 1950s, colistin was first used in clinical practice in the United States in 1959 [5]. The IV formulation is approved by the Food and Drug Administration (FDA) for use in the US; however, its aerosolized use is not. Despite this, aerosolized colistin is regularly used in combination with IV CMS and/or other antibiotics for its perceived synergistic benefits in the management of hospital-associated (HAP) or ventilator-associated pneumonia (VAP) caused by MDROs (frequently *K. pneumoniae, P. aeruginosa, A. baumannii,* and *Enterobacter* spp.) [6,7,8,9]. However, at the time of FDA approval, colistin did not undergo the standard rigorous pharmacokinetic (PK) and safety studies—which are now mandatory.

Several studies have suggested that a steady-state colistin concentration of 2 mg/L is a reasonable target when initiating therapy, and that this can be achieved by every 12 h dosing [10,11,12]. The FDA recommends a daily dose of 2.5–5 mg/kg of colistin base activity divided into two to four equal doses to achieve this level in subjects with creatinine clearance ≥ 80 mL/min [11,12]. However, the optimal colistin dosing remains unclear. Single once-daily dosing is associated with greater toxicity and the emergence of drug resistance [1,13], as is an extended duration of treatment [14,15,16]. Additionally, PK data regarding colistin are scarce in certain populations (e.g., intensive care patients vs. cystic fibrosis) and tend to vary between them [2]. Thus, there is an urgent need for the clarification of the safe use of CMS, including in its aerosolized form, and whether intravenous administration significantly adds to aerosolized colistin in achievable lung concentrations for the treatment of pneumonia.

The aim of the present study was to determine whether the total daily dose of colistin could be safely increased to 7.5 mg/kg ideal body weight by dosing every 8 h (q8h) rather than every 12 h (q12h), and to characterize the PK and safety and tolerability of CMS after intravenous (IV) and aerosolized administration of CMS in healthy adults with normal kidney function.

## 2. Results

### 2.1. Demographic and Other Baseline Characteristics

A summary of the study cohort and dosing design is presented in Table 1, while the subject demographic and other baseline characteristics in the PK and safety populations is presented in Table 2. Except for gender, demographics in both populations were well matched across cohorts based on age, race, and ethnicity.

### 2.2. Pharmacokinetic Parameters of Colistin A, Colistin B, CMS A, and CMS B

**Figure 1:**
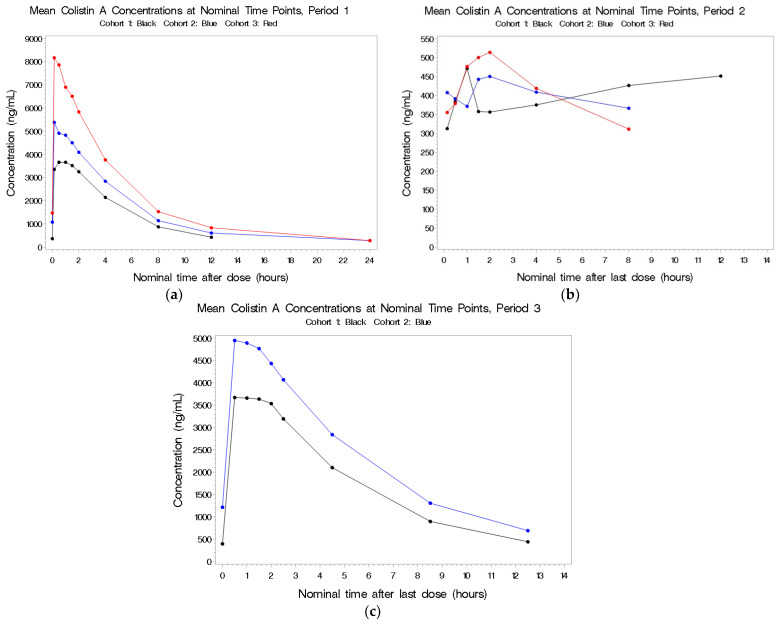
(**a**–**c**) Mean plasma concentrations for colistin A after last dose in Period 1 by cohort.

**Figure 2:**
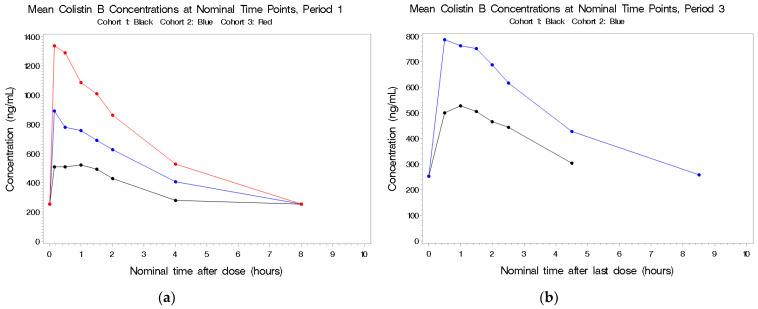
(**a**,**b**) Mean plasma concentrations for colistin B after last dose in Period 1 by cohort.

**Figure 3:**
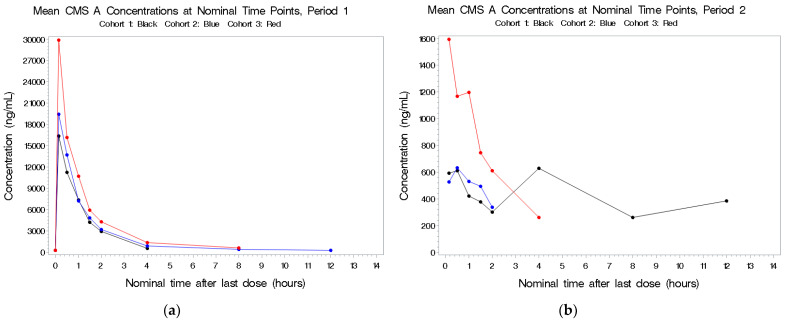

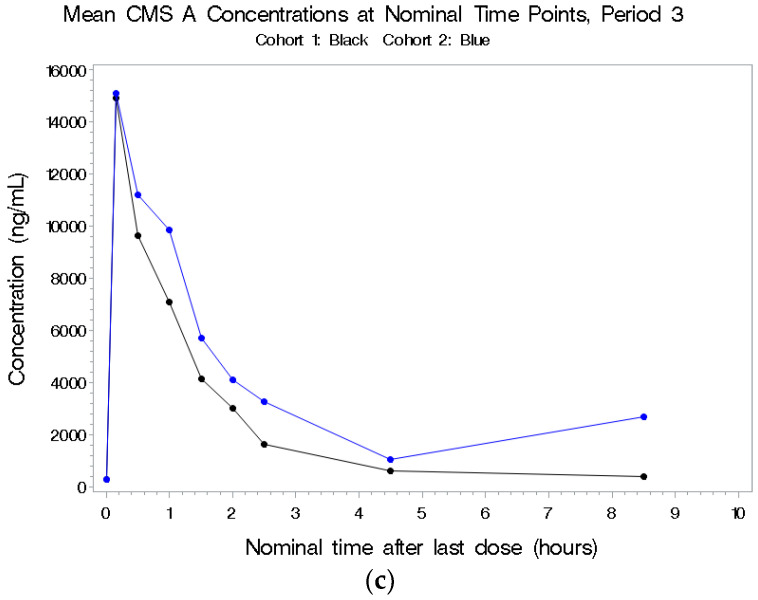
(**a**–**c**) Mean plasma concentrations for CMS A after last dose in Period 1 by cohort.

**Figure 4:**
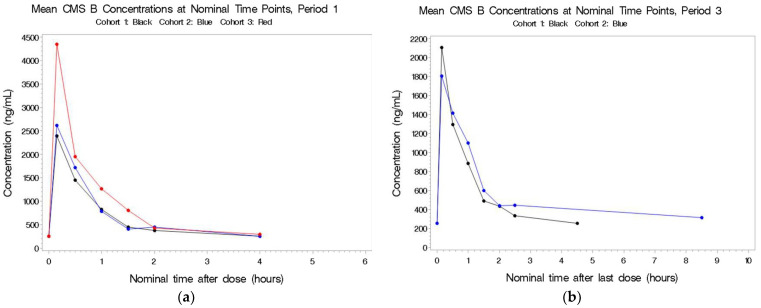
(**a**,**b**) Mean plasma concentrations for CMS B after last dose in Period 1 by cohort.

The PK population consisted of 29 subjects in total. In Cohort 1, 9 subjects completed all three treatment periods, 8 subjects in Cohort 2 completed all three periods, whereas for Cohort 3, only 7 of the 12 subjects were dosed in Period 2 and only 6 were dosed in Period 3 (Table 2). Plots of the individual plasma concentrations of the four colistin analytes by period and cohort are presented in Figure 1, Figure 2, Figure 3 and Figure 4, whereas comparisons of dosing type and analytes for each cohort are presented in Supplementary Tables S1–S10.

#### 2.2.1. Colistin A

See Figure 1a–c. Following IV exposure in Periods 1 and 3, circulating concentrations were dose-related but not dose-proportional. As the total dose of colistin increased from 5 to 10 mg/kg, there was a corresponding 1.6-fold increase in both total colistin A exposure (AUC(0–t)) and peak colistin A concentration (Cmax). The range of individual Tmax values was similar for all three cohorts. The t1/2, CL, and Vdss values for colistin A were consistent across the three cohorts. Circulating colistin A concentrations following aerosol dosing were low and not related to the dose administered. The combination of IV and aerosol administration did not increase the circulating concentrations compared to IV dosing alone.

#### 2.2.2. Colistin B

See Figure 2a,b. Following IV exposure in Periods 1 and 3, circulating concentrations were dose-related but not dose-proportional. As the total colistin dose increased from 5 to 10 mg/kg, there was an approximate 1.4-fold increase in colistin B Cmax and a 1.3-fold increase in colistin B AUC(0–t). Plasma concentrations following IV dosing in Period 1 and combined IV and aerosolized dosing in Period 3 were comparable. There was no accumulation of this drug and no detectable concentrations following aerosol administration alone. As with colistin A, the elimination t1/2 was about 3.5 h.

#### 2.2.3. CMS A

See Figure 3a–c. There were higher concentrations in plasma following combined IV and aerosolized administration in Period 3 compared to IV dosing alone in Period 1 in the first cohort. The half-life was short (~1 h), and there was no indication of accumulation of this metabolite. CMS A was detectable following aerosol-only administration in a dose-related manner; for the 1.5-fold increase in total CBA dose from 500 to 750 mg (based on 70 kg), there was no increase in CMS A Cmax and an approximate 1.3-fold increase in CMS A AUC(0–inf).

#### 2.2.4. CMS B

See Figure 4a,b. IV administration during Periods 1 and 3 resulted in very similar concentrations of CMS B, indicating that aerosol did little to contribute to the circulating concentrations. CMS B was not detectable in Cohorts 1 and 2 following aerosol-only administration.

### 2.3. Comparison of Colistin Analyte Concentrations in Plasma and BAL

**Figure 5:**
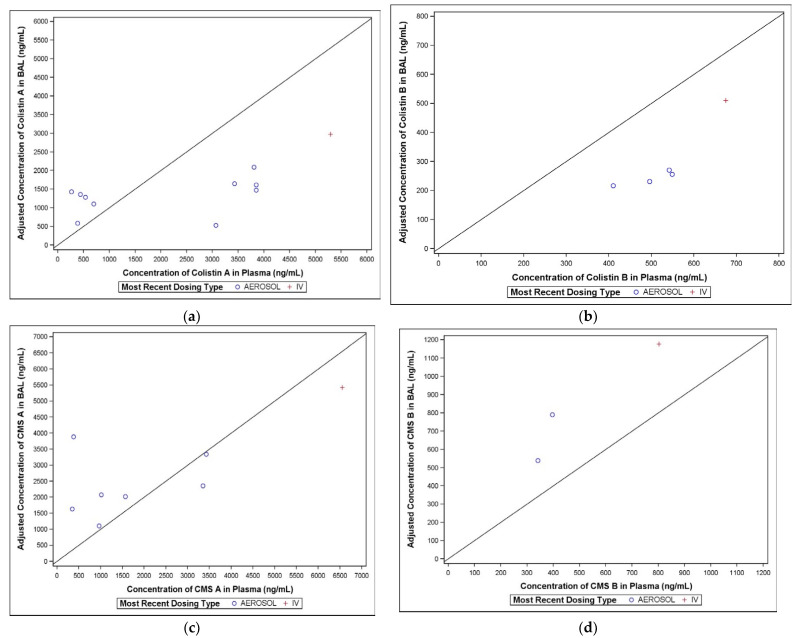
(**a**–**d**) Comparison of colistin A concentrations in BAL and plasma.

Plots comparing the adjusted colistin concentration in BAL to the corresponding observed plasma concentrations are presented in Figure 5a–d.

#### 2.3.1. Colistin A

Only one of the BAL samples following IV dosing in Period 1 contained quantifiable concentrations of colistin A, compared with all thirteen BAL samples following aerosol administration in Period 2. The BAL colistin A concentrations ranged from 323 to 2160 ng/mL (Figure 5a).

#### 2.3.2. Colistin B

Only one of the BAL samples following IV dosing in Period 1 contained quantifiable colistin B concentrations, compared with eleven of the thirteen BAL samples in Period 2. The BAL colistin B concentrations ranged from BQL to 324 ng/mL (Figure 5b).

#### 2.3.3. CMS A

Following aerosol administration in Period 2, 12 of the 13 BAL samples contained quantifiable concentrations of CMS A, compared with only 1 of the BAL samples following IV dosing in Period 1. The BAL CMS A concentrations ranged from BQL to 3096 ng/mL (Figure 5c).

#### 2.3.4. CMS B

Similar to CMS A, only one of the BAL samples following IV dosing in Period 1 contained quantifiable concentrations of CMS B, compared with eleven of the thirteen BAL samples in Period 2. The BAL CMS B concentrations ranged from BQL to 769 ng/mL (Figure 5d).

### 2.4. Subject Safety and Tolerability

The safety population consisted of 31 subjects: 9, 10, and 12 to Cohorts 1, 2, and 3, respectively (Table 2). The mean age across cohorts ranged from 23.9 to 25.2 years, race was primarily white, and the majority of subjects were male in Cohorts 1 and 3 and female for Cohort 2.

A summary of treatment-emergent adverse events (TEAEs) by period and treatment modality is presented in Table 3. The overall proportion of subjects who experienced any TEAEs during any period was 30/31 (96.8%). A total of 287 AEs were reported. The majority (138/287, 48.1%) occurred during Period 1. Most of the events were mild or moderate in severity and related to the study drug; the distribution across cohorts was similar. There was one serious TEAE of acute kidney injury that resulted in the discontinuation of the subject from the study. There were no deaths in the study.

### 2.5. Nephrotoxicity and Kidney Biomarkers as Indicators of Future Nephrotoxicity

Increasing the colistin dose from 5 mg/kg/day (Cohort 1; 2.5 mg/kg q12h) to 7.5 mg/kg/day (Cohort 2; 2.5 mg/kg q8h) did not result in increases in serum creatinine. The mean creatinine did not differ between subjects in Cohorts 1 and 2 and for dosing periods 2 and 3 for Cohort 3. However, the reversible occurrence of nephrotoxicity (as indicated by elevated serum creatinine at the 24 h post first dose time point) was observed in four subjects in Cohort 3 after receiving three doses of IV colistin 2.5 mg/kg q8h (serum creatinine increases of 0.67/95.7%, 0.82/82.0%, 0.69/73.4%, and 0.3/45.5%, respectively). The events resulted in the discontinuation of the study drug and discontinuation of the subjects from the study. The increases in creatine resolved over 10 days (±4 days).

To assess the usefulness of kidney function biomarkers in indicating future nephrotoxicity, Spearman correlation coefficients were calculated for serum kidney biomarkers cystatin, β-microglobulin, and NGAL with the serum creatinine values. Before the first dose and early time points after the first dose were used for the biomarkers, and 24 h post first dose creatinine values were used. The correlation coefficients and the respective p values are shown in Table 4. Correlation coefficients did not reach statistical significance (all *p* values > 0.05), nor were the coefficients nominally high enough to indicate clinically meaningful relationships. The biomarkers did not seem to be useful in indicating subsequent nephrotoxicity in Cohort 3 subjects; no correlation of the measured biomarkers for acute kidney injury and serum creatinine measurement was noted.

### 2.6. Neurotoxicity

Seven of eighteen subjects (Periods 1 and 3, combined) in Cohort 1 (39%) who received the standard dose of colistin (2.5 mg/kg q12h × 2 doses) and 18/20 (90%) Cohort 3 subjects who got the higher dose (3.3 mg/kg q8h × 3 doses) reported perioral paresthesias that were self-limited (Table 3). Paresthesias were described as “numbness” or “tingling” at the tip of the tongue; they often evolved to become circumoral and/or involve the cheeks and chin, and occasionally the fingertips. They resolved over 6–8 h, and subjects remained symptom free.

There was no ataxia reported in Cohort 1; however, 7 subjects in Cohort 2 and 10 subjects in Cohort 3 experienced ataxia that occurred during Period 1 (IV administration) and/or Period 3 (aerosol + IV administration) (Table 3). Based on the SARA scores (Table 5), the events were primarily mild in severity (mainly affected gait), occurred within a day of study drug administration, and were considered related to the study drug. Most of the events resolved within 24 h. One subject’s ataxia resulted in the discontinuation of the study drug and was considered moderate in severity, lasted 2 days, and occurred in Period 1, one day after study drug administration.

## 3. Discussion

This is the first phase 1 trial to assess the PK and safety of CMS. The trial sought to determine whether every 8-hourly dosing of CMS would result in greater plasma concentrations of the active drug (colistin A and B) than the standard every 12-hourly dosing without increasing nephrotoxicity. Furthermore, it examined whether aerosolized CMS would provide a greater concentration of the active drug to the lungs (as measured using BAL) than IV administered CMS alone or in combination with aerosolized administration. Both trial objectives were met.

Every 8-hourly dosing of CMS resulted in a 33% increase in serum concentrations of colistin A, the major active component of CMS, without any evidence of increased nephrotoxicity. These results suggest that the non-enzymatic hydrolysis of CMS to active colistin is rate-limiting, and that once all, or most of the CMS has been hydrolyzed, it is safe to introduce more. CMS is cleared through the kidneys, whereas colistin is re-absorbed [3]. If, as seems likely, nephrotoxicity results primarily from renal clearance of CMS [4], allowing for full hydrolysis of CMS between doses should be safe, as there would be no accumulation of CMS.

Not only did every 8-hourly dosing regimen not result in any increase in creatinine, there also was no evidence of subtle nephrotoxicity, as judged by the absence of any increases in the three biomarkers of kidney damage that we measured. Colistin, on the other hand, is reabsorbed and would be expected to accumulate. Every 8-hourly dosing not only resulted in an increase in colistin Cmax but in maintenance of colistin concentrations above the target of 2 μg/mL for 6 of the 8 h between doses.

Every 8-hourly dosing regimen was, however, associated with greater neurotoxicity. Colistin toxicity produces a wide array of neurological symptoms, with paresthesias being the most frequently encountered [18]. Thus, while lingual and perioral paresthesias were not unexpected, the degree of ataxia was. The paresthesias were bothersome but did not cause any disability and resolved over several hours. The ataxias were mildly to moderately disabling, as they primarily affected gait. Why ataxias were seen only during Period 3 dosing of Cohort 2 and not during Period 1 is unexplained, as the IV dosing schedule was the same in both periods, and there was ample time for washout between the periods. We can only speculate that colistin and/or CMS may bind to neural tissues, persist for much longer than in renal tissues, and accumulate over time. The ataxias, however, did not persist, which speaks against this hypothesis. Thus, the usefulness of every 8-hourly dosing regimen in increasing plasma colistin concentrations without concomitant nephrotoxicity will need to be balanced against the increase in neurotoxicity. This observation may have implications in critically ill hospitalized patients who are often mechanically ventilated and sedated, in whom neurotoxicity would be difficult to detect when present.

The aerosolized administration of CMS provided little and/or no additional plasma exposure to colistin A or B, and vice versa. Following the aerosolized administration of CMS, BAL samples had quantifiable concentrations of colistin A and B (range 323 to 2160 ng/mL). Thus, aerosolized CMS can deliver high concentrations of an active drug directly to the site of the infection without significant risk of nephrotoxicity and neurotoxicity, while simultaneously overcoming the challenge of IV administration not achieving adequate concentrations in the lung parenchyma, especially in HAP/VAP caused by MDROs [19,20].

Whether the aerosolized administration of CMS translates into greater drug efficacy against MDROs in the management of HAP/VAP caused by MDROs, however, still remains a matter of controversy, as is the issue of the efficacy of colistin combination therapy. A retrospective study of pneumonia patients who received aerosolized colistin therapy for a median of 14 days reported the microbiological eradication of the causative MDRO (A. baumannii or P. aeruginosa) in 18 out of 21 cases [21]. This high rate of eradication corroborates another retrospective study in which 37 out of 40 patients who received aerosolized colistin therapy for A. baumannii or P. aeruginosa infection had negative follow-up cultures [21]. Similarly, a recent prospective study of colistin–meropenem combination versus colistin monotherapy showed a significant decrease in mortality (16.7% (5/30) vs. 43.3% (13/30); *p* = 0.047) in the treatment of HAP/VAP due to MDR K. pneumoniae [7]. However, the AIDA Trial—a multi-country, open-label, randomized, controlled trial and the largest to date—recently failed to demonstrate the superiority of colistin combination therapy over monotherapy. Of the 406 patients enrolled, the majority had pneumonia or bacteremia (355/406, 87%) and A. baumannii was the most common causative organism (312/406, 77%). Combination therapy with meropenem and colistin was not superior to colistin monotherapy at 14 days in reducing mortality [22].

In their totality, these studies suggest that aerosolized colistin therapy is associated with favorable outcomes in certain populations. However, the populations studied are mostly small; patients often have co-infections with other pathogens and have significant comorbidities. When aggregated together, favorable trends may not hold true [23]. The potential for aerosolized CMS to be used as adjunctive therapy is, however, promising, as shown by our study and others [6,7,8,9,21,23,24,25]. The establishment of standardized dosing and indications for aerosolized CMS will be extremely helpful as research continues in this area, due to an already heterogeneous target population.

Our study had a few limitations. All study participants were healthy subjects restricted to a single study site; study results may therefore not be generalizable to other populations who may be healthy but at risk for infections (cystic fibrosis, frequent urinary tract infections, and prior antibiotic exposure). Additionally, the small size of the study population presents a difficulty in generalizing study results to a wider population.

## 4. Materials and Methods

### 4.1. Materials

Colistimethate for Injection, USP, from X-Gen Pharmaceuticals, Inc. (Horsehead, NY, USA) was utilized as lyophilized cakes in individual vials equivalent to 150 mg of colistin base activity per vial.

### 4.2. Trial Design, Study Population, and Ethics Approval

This was a single site, open-label, prospective study in 39 healthy adult volunteers aged 18 to 45 years inclusive and was conducted from August 2013 to 4 May 2017 (registered at Clinicaltrials.gov, registration number NCT01863719). Full subject eligibility (inclusion and exclusion) criteria are provided in Supplementary Materials. All study procedures were conducted at Case Western Reserve University and University Hospitals Cleveland Medical Center (UHCMC) in Cleveland, Ohio. The study was approved by the Institutional Review Board at UHCMC, and all subjects provided written informed consent prior to participating in the study.

### 4.3. Study Objectives

The primary objective of the study was to evaluate the safety and tolerability of multiple doses of aerosolized or IV CMS, separately or in combination, in healthy adult subjects. The secondary objective was to determine the pharmacokinetics of multiple doses of aerosolized or IV CMS, separately or in combination, in healthy adult subjects through the measurement of plasma concentrations. The exploratory objective was to evaluate for acute kidney injury in healthy subjects receiving IV and aerosolized CMS with selected serum and urine biomarkers.

### 4.4. Methodology

The study consisted of 4 cohorts: 9 subjects each in Cohorts 1, 2, and 3, and 12 subjects in Cohort 4 (Table 1). Cumulative safety data for the primary and secondary objectives were reviewed by the Safety Monitoring Committee after Cohorts 1, 2, and 3. Due to safety concerns, Cohort 4 was not initiated.

During dosing Period 1, subjects in Cohorts 1, 2, and 3 received 2 to 3 doses of IV CMS (total exposure 5–10 mg/kg) based on the specific cohort regimen. During dosing Period 2, the same subjects then received 2 to 4 doses of 75 mg of CMS via aerosolized administration, and during dosing Period 3, subjects received a combination of IV and aerosolized CMS based on the specific cohort regimen. Each dosing period was followed by a washout period of at least 3 days (Table 1).

Within each cohort, 3 randomly selected subjects underwent a research bronchoscopy and bronchoalveolar lavage (BAL) per dosing after the completion of all dosing for that period such that no subject underwent BAL more than once.

### 4.5. Pharmacokinetic Assessments

#### 4.5.1. Plasma Samples

For all plasma PK collections, 3 mL samples of whole blood were drawn into collection tubes containing sodium heparin and placed immediately on ice at specified time intervals. Post-dose samples 30 min to 4 h had a ±10 min window; post-dose samples > 4 to 8 h had a ±15 min window; and samples > 8 h had a ±30 min window (please see Supplementary Data S2 for full PK dosing schedule). The blood was centrifuged at 2500× *g* within 2 h of collection, and the plasma fraction was removed and frozen at −70 °C for shipment to the Analytical Pharmacology Laboratory at the University of Toledo. For each sample, total plasma concentrations of four colistin analytes were measured: (1) colistin A; (2) colistin B; (3), CMS A; (4) CMS B.

#### 4.5.2. Bronchoalveolar Lavage Samples

BAL specimens were obtained from 3 subjects per dosing period 3 h (±2 h) after the completion of both doses in dosing Periods 1 and 2 and all doses in dosing Period 3. The concentrations of the four colistin analytes were measured as above.

### 4.6. Kidney Biomarkers

Serum samples for kidney biomarkers were obtained for serum cystatin C, NGAL, and serum β microglobulin levels. Urine samples were obtained for urine protein, creatinine, and microalbumin excretion (see Supplementary Tables S1–S10 and Data S1–S6).

### 4.7. Pharmacokinetic Analysis

The PK analysis was conducted using Phoenix™ WinNonlin^®^ Version 6.2 (Pharsight, a Certara Company). In all matrices (plasma or BAL), the concentrations of colistin A and colistin B were measured. The concentrations of CMS A and CMS B were then determined indirectly. The CMS A concentrations were calculated by subtracting the concentrations of colistin A determined in the samples before hydrolysis from the colistin A concentrations determined after hydrolysis with sulfuric acid, which converts CMS A into colistin A. The colistin A concentration determined to be the hydrolysis product of CMS A was then multiplied by the molar equivalency to yield the concentration of CMS A. The same procedure was repeated for CMS B.

### 4.8. Bioanalytical Methods

Concentration versus time profiles were investigated for each of the four colistin analytes. These profiles were augmented by the inclusion of estimated concentrations at additional time points, including dosing Period 1—immediately prior to the final IV dose; dosing Period 3—immediately prior to the final IV dose; and after the final IV dose. The following PK parameters were estimated for each: maximum observed concentration (Cmax), area under the plasma concentration versus time curve, AUC (e.g., AUC 0–inf), AUC 0–t) versus time curve, time to maximum observed concentration (Tmax), elimination half-life (t1/2), slope of the post-distributive terminal portion of plasma concentration versus time curve (Kel), total body clearance/bioavailability (CL/F), and the apparent steady-state volume of distribution/bioavailability (Fdss/F). The concentrations of intravenously administered CMS, aerosolized drug, and both delivery methods used simultaneously were compared. In addition, the presence or absence of a proportionality dose-dependent effect was determined.

### 4.9. Safety Assessments

Safety assessments for TEAEs were conducted, including physical examinations (including neurological evaluations), physical examinations (including neurologic evaluation), vital signs, clinical laboratory tests, selected biomarkers for acute kidney injury (see section on kidney biomarkers), spirometry, and ECG measurements (see Supplementary Data S5 and S6 for clinical laboratory evaluations and schedule of events). If symptoms of ataxia were present, SARA (Scale for the Assessment and Rating of Ataxia, according to Schmitz-Hübsch et al. [17]) assessment forms were completed to assess their severity.

## 5. Conclusions

In summary, the IV administration of CMS did not increase lung concentrations of active colistin compared with the administration of aerosolized CMS alone and vice versa. Every 8-hourly dosing resulted in a 33% increase in the mean serum colistin A and colistin B concentrations. There was no increase in nephrotoxicity except in a few subjects, but the q8h dosing did result in an increase in neurotoxicity, primarily perioral and lingual paresthesias and self-limited ataxia. These findings call into question the utility of the widespread use of colistin combination therapy, especially in the treatment of pneumonia caused by MDR bacteria. On the other hand, aerosolized therapy may be beneficial as an adjunctive therapy with other novel antimicrobials; this requires further investigation.

## Figures and Tables

**Table 1:** Study design.

Cohort	N	Dosing Period 1 (IV)	Washout	Dosing Period 2 (aerosolized)	Washout	Dosing Period 3 (combination IV and aerosolized)
1	9	2.5 mg/kg colistin base activity q12h × 2 doses (total exposure: 5 mg/kg)	At least 3 days	75 mg colistin base activity q12h × 2 doses	At least 3 days	IV: 2.5 mg/kg colistin base activity q12h × 2 doses Aerosol: 75 mg colistin base activity q12h × 2 doses
2	9	2.5 mg/kg colistin base activity q8h × 3 doses (total exposure: 7.5 mg/kg)	At least 3 days	75 mg colistin base activity q8h × 3 doses	At least 3 days	IV: 2.5 mg/kg colistin base activity q8h × 3 doses Aerosol: 75 mg colistin base activity q8h × 3 doses
3	9	3.3 mg/kg colistin base activity q8h × 3 doses (total exposure: 10 mg/kg)	At least 3 days	75 mg colistin base activity q6h × 4 doses	At least 3 days	IV: 3.3 mg/kg colistin base activity q8h × 3 doses Aerosol: 75 mg colistin base activity q6h × 4 doses

h = hour, IV = intravenous. Note: Subjects were inpatients throughout each dosing period and remained inpatients for 24 h after receiving their last dose in each dosing period.

**Table 2:** Pharmacokinetic and safety populations.

Characteristics	Pharmacokinetic Population			Safety Population		
	Cohort 1 (N = 9)	Cohort 2 (N = 8)	Cohort 3 (N = 12)	Cohort 1 (N = 9)	Cohort 2 (N = 10)	Cohort 3 (N = 12)
Age (years)						
n	9	8	12	9	10	12
Mean (SD)	23.9 (4.4)	24.9 (4.0)	24.9 (5.1)	23.9 (4.4)	25.2 (4.7)	24.9 (5.1)
Median	21.0	23.5	22.5	21.0	23.5	22.5
Min, Max	19, 30	21, 31	19, 38	19, 30	20, 33	19, 38
Gender						
Male	6 (66.7%)	2 (25.0%)	8 (66.7%)	6 (66.7%)	3 (30.0%)	8 (66.7%)
Female	3 (33.3%)	6 (75.0%)	4 (33.3%)	3 (33.3%)	7 (70.0%)	4 (33.3%)
Unknown	0	0	0	0	0	0
Race						
White	7 (77.8%)	6 (75.0%)	8 (66.7%)	7 (77.8%)	7 (70.0%)	8 (66.7%)
Black/African American	0	1 (12.5%)	1 (8.3%)	0	2 (20.0%)	1 (8.3%)
American Indian/Alaskan Native	0	0	0	0	0	0
Native Hawaiian /Other Pacific Islander	0	0	0	0	0	0
Asian	2 (22.2%)	1 (12.5%)	3 (25.0%)	2 (22.2%)	1 (10.0%)	3 (25.0%)
Other	0	0	0	0	0	0
Ethnicity						
Hispanic or Latino	1 (11.1%)	0	2 (16.7%)	1 (11.1%)	0	2 (16.7%)
Non-Hispanic or Latino	8 (88.9%)	8 (100.0%)	10 (83.3%)	8 (88.9%)	10 (100.0%)	10 (83.3%)

max = maximum, min = minimum, N/n = number, SD = standard deviation. Note: Percentages are based on the total number of subjects in the indicated cohort.

**Table 3:** Treatment-emergent adverse events ≥ 10% overall by preferred term—safety population.

Characteristics	Cohort 1 (N = 9)	Cohort 2 (N = 10)	Cohort 3 (N = 12)	Overall (N = 31)
Total Number of Adverse Events	60	108	119	287
Number of Subjects with at Least One Adverse Event	9 (100.0%)	10 (100.0%)	11 (91.7%)	30 (96.8%)
Investigations	7 (77.8%)	6 (60.0%)	11 (91.7%)	24 (77.4%)
Blood calcium decreased	2 (22.2%)	0	4 (33.3%)	6 (19.4%)
Blood creatinine increased	0	1 (10.0%)	4 (33.3%)	5 (16.1%)
Lymphocyte count decreased	2 (22.2%)	1 (10.0%)	1 (8.3%)	4 (12.9%)
Prothrombin time prolonged	3 (33.3%)	3 (30.0%)	1 (8.3%)	7 (22.6%)
Red blood cells urine	0	1 (10.0%)	3 (25.0%)	4 (12.9%)
White blood cells urine positive	0	2 (20.0%)	7 (58.3%)	9 (29.0%)
Nervous system disorders	6 (66.7%)	10 (100.0%)	11 (91.7%)	27 (87.1%)
Ataxia	0	7 (70.0%)	10 (83.3%)	17 (54.8%)
Dizziness	0	3 (30.0%)	2 (16.7%)	5 (16.1%)
Headache	5 (55.6%)	5 (50.0%)	4 (33.3%)	14 (45.2%)
Paresthesia	5 (55.6%)	10 (100.0%)	9 (75.0%)	24 (77.4%)
Renal and urinary disorders	1 (11.1%)	2 (20.0%)	7 (58.3%)	10 (32.3%)
Glycosuria	0	0	5 (41.7%)	5 (16.1%)
Proteinuria	1 (11.1%)	2 (20.0%)	7 (58.3%)	10 (32.3%)
Respiratory, thoracic and mediastinal disorders	3 (33.3%)	3 (30.0%)	2 (16.7%)	8 (25.8%)
Cough	1 (11.0%)	3 (30.0%)	2 (16.7%)	6 (19.4%)

N = number. Note: The total number of adverse events counts all adverse events for subjects. Subjects may have more than one adverse event per body system and preferred term. At each level of subject summarization, a subject was counted once if they reported one or more events. Percentages are based on the total number of subjects.

**Table 4:** Spearman correlations of serum biomarkers with serum creatinine.

Time Point	Biomarker	Correlation Coefficient	*p*-Value
Before First Dose	Cystatin	0.15	0.63
	Microglobulin	0.32	0.34
	NGAL	−0.25	0.43
0.5 hr After First Dose	Cystatin	0.03	0.93
	Microglobulin	0.25	0.49
	NGAL	−0.26	0.43

NGAL, neutrophil gelatinase-associated lipocalin.

**Table 5:** Neurotoxicity as assessed by the Scale for the Assessment and Rating the of Ataxia (SARA) method [17].

SARA Test Parameter	Subject SARA Scores ^1^				
	20012078	20012079	20012080	20012081	20012082
Gait ^2^	1	2	3	1	1
Stance ^3^	0	2	2	0	1
Sitting ^4^	0	0	0	0	0
Speech disturbance ^4^	0	0	0	0	0
Finger chase—right ^5^	0	0	0	0.5	0
Finger chase—left ^5^	0	0.5	0	0	0
Nose–finger—right ^6^	0	0	0.5	0	0
Nose–finger—left ^6^	0	0	0.5	0	0
Fast alternating hand movements—right ^4^	0	0	0	0	0
Fast alternating hand movements—left ^4^	0	0	0	0	0
Heel–shin slide—right ^4^	0	0	0	0	0
Heel–shin slide—left ^4^	0	0	0	0	0
Cumulative SARA Score	1	4.5	6	1.5	2

^1^ Based on the highest score for any visit; ^2^ gait score key: 0 = normal, able to stand in tandem for >10s; 1 = able to stand with feet together without sway, but not in tandem for >10s; 2 = able to stand with feet together for >10s, but only with sway; ^3^ stance score key: 0 = normal, able to stand in tandem for >10s; 1 = able to stand with feet together without sway, but not in tandem for >10s; 2 = able to stand with feet together for >10s, but only with sway; ^4^ sitting, speech disturbance, fast alternating hand movements, and heel–shin slide score key: 0 = normal; ^5^ finger chase score key: 0 = normal, no dysmetria, 0.5 = dysmetria, under/overshooting target < 5 cm (unilateral), 1 = dysmetria, under/overshooting target < 5 cm (bilateral); ^6^ Nose–finger score key: 0 = no tremor; 0.5 = tremor with an amplitude < 2 cm (unilateral); 1 = tremor with an amplitude < 2 cm (bilateral).

## Data Availability

All supporting data are presented in the manuscript and in the Supplementary Materials.

## Supplementary Materials

The following are available online at https://www.mdpi.com/article/10.3390/antibiotics11040490/s1. Table S1–S10: Tables of PK parameters of colistin analytes, Supplementary Data S1: Diagnosis and Criteria for Inclusion and Exclusion. Supplementary Data S2: Pharmacokinetic Sampling Schedule, Supplementary Data S3: Serum Kidney Biomarker Time Points, Supplementary Data S4: Urine Sample for Kidney Biomarkers, Supplementary Data S5: Clinical Laboratory Evaluations, Supplementary Data S6: Schedule of Events—Cohorts 1 Through 4, All Dosing Periods,
