# Supplementary material for: Dosing Colistimethate Every 8 h Results in Higher Plasma Concentrations of Active Colistin Than Every 12-Hourly Dosing without Increase in Nephrotoxicity: A Phase 1 Pharmacokinetics Trial in Healthy Adult Volunteers
Source: Antibiotics (Basel). 2022 Apr 6;11(4):490. doi: 10.3390/antibiotics11040490 (PMC9029538; doi:10.3390/antibiotics11040490)
Supplement: Supplementary file 1 [file antibiotics-11-00490-s001.zip › antibiotics-1631573-supplementary.pdf]

Supplementary Materials

Table S1. Mean (SD) Plasma PK Parameters for Colistin A in Period 1 by Cohort

Parameter	Cohort 1	Cohort 2	Cohort 3
C_{max} (ng/mL)	3950 (331)	5360 (765)	8740 (2190)
t_{max} (h)[a]	1.02 (0.583 – 2.00)	0.600 (0.533 – 2.00)	1.00 (0.500 – 2.00)
$AUC_{(0-t)}$ (h*ng/mL)	21800 (2400)	30400 (7580)	49100 (14200)
$AUC_{(0inf)}$ (h*ng/mL)	23800 (2710)	33000 (7530)	52400 (15700)
$t_{1/2}$ (h)	3.32 (0.158)	3.47 (0.766)	4.22 (1.64)
CL (L/h)	6.16 (0.801)	4.47 (0.865)	3.87 (1.00)
Vdss (L)	29.5 (4.18)	21.7 (3.19)	22.2 (4.84)

Cohort 1 = IV: 2.5 mg/kg colistin base activity q12h x 2 doses (total exposure: 5 mg/kg).
Cohort 2 = IV: 2.5 mg/kg colistin base activity q8h x 3 doses (total exposure: 7.5 mg/kg).
Cohort 3 = IV: 3.3 mg/kg colistin base activity q8h x 3 doses (total exposure: 10 mg/kg).
PK Parameters were based on the serial plasma collections taken after the administration of the final dose during the study period.
a. median (min-max).

Table S2. Mean (SD) Plasma PK Parameters for Colistin A in Period 2 by Cohort

Parameter	Cohort 1	Cohort 2	Cohort 3
C_{max} (ng/mL)	493 (169)	464 (162)	553 (260)
t_{max} (h)[a]	3.38 (1.50 – 4.32)	2.33 (1.83 – 4.47)	1.42 (1.27 – 2.35)
$AUC_{(0-t)}$ (h*ng/mL)	4220 (2150)	2260 (1530)	2860 (2040)
$AUC_{(0inf)}$ (h*ng/mL)	8170 (-)	8240 (638)	16400 (14500)
$t_{1/2}$ (h)	8.37 (-)	7.81 (1.33)	26.0 (31.2)
CL/F (L/h)	8.17 (-)	8.12 (0.628)	6.45 (4.28)
Vdss/F (L)	98.7 (-)	92.1 (22.7)	130 (58.7)

Cohort 1 = Aerosol: 75 mg colistin base activity q12h x 2 doses.
Cohort 2 = Aerosol: 75 mg colistin base activity q8h x 3 doses.
Cohort 3 = Aerosol: 75 mg colistin base activity q6h x 4 doses.
PK Parameters were based on the serial plasma collections taken after the administration of the final dose during the study period.
a. median (min-max).

Table S3. Mean (SD) Plasma PK Parameters for Colistin A in Period 3 by Cohort

Parameter	Cohort 1	Cohort 2	Cohort 3
C_{max} (ng/mL)	3980 (824)	5070 (1330)	NR
t_{max} (h)[a]	1.43 (0.883 – 2.38)	1.28 (0.767 – 1.95)	NR
$AUC_{(0-t)}$ (h*ng/mL)	22800 (4760)	33200 (12600)	NR
$AUC_{(0inf)}$ (h*ng/mL)	25200 (4970)	36000 (12500)	NR
$t_{1/2}$ (h)	3.73 (0.735)	4.22 (0.965)	NR
CL/F (L/h)	5.90 (0.896)	4.32 (1.35)	NR
Vdss/F (L)	32.0 (9.89)	25.1 (5.19)	NR

NR = Summary parameters not reported for Cohort 3.
Cohort 1 = IV: 2.5 mg/kg colistin base activity q12h x 2 doses, Aerosol: 75 mg colistin base activity q12h x 2 doses.
Cohort 2 = IV: 2.5 mg/kg colistin base activity q8h x 3 doses, Aerosol: 75 mg colistin base activity q8h x 3 doses.
Cohort 3 = IV: 3.3 mg/kg colistin base activity q8h x 3 doses, Aerosol: 75 mg colistin base activity q6h x 4 doses.
PK Parameters were based on the serial plasma collections taken after the administration of the final dose during the study period.
a. median (min-max).

Table S4. Mean (SD) Plasma PK Parameters for Colistin B in Period 1 by Cohort

Parameter	Cohort 1	Cohort 2	Cohort 3
C_{max} (ng/mL)	570 (58.3)	891 (148)	1390 (404)
t_{max} (h)[a]	0.667 (0.583 – 2.00)	0.600 (0.533 – 2.00)	0.600 (0.500 – 1.00)
$AUC_{(0-t)}$ (h*ng/mL)	1940 (528)	3590 (1160)	5680 (2010)
$AUC_{(0inf)}$ (h*ng/mL)	3290 (1060)	5150 (1050)	7160 (2100)
$t_{1/2}$ (h)	3.45 (1.74)	3.64 (0.619)	3.55 (1.02)
CL (L/h)	5.81 (1.39)	3.54 (0.792)	3.49 (0.868)
Vdss (L)	26.3 (5.23)	18.1 (2.37)	17.3 (3.85)

Cohort 1 = IV: 2.5 mg/kg colistin base activity q12h x 2 doses (total exposure: 5 mg/kg).
Cohort 2 = IV: 2.5 mg/kg colistin base activity q8h x 3 doses (total exposure: 7.5 mg/kg).
Cohort 3 = IV: 3.3 mg/kg colistin base activity q8h x 3 doses (total exposure: 10 mg/kg).
PK Parameters were based on the serial plasma collections taken after the administration of the final dose during the study period.
a. median (min-max).

Table S5. Mean (SD) Plasma PK Parameters for Colistin B in Period 3 by Cohort

Parameter	Cohort 1	Cohort 2	Cohort 3
C_{max} (ng/mL)	577 (118)	806 (212)	NR
t_{max} (h)[a]	1.57 (0.883 – 2.38)	1.17 (0.883 – 1.73)	NR
$AUC_{(0-t)}$ (h*ng/mL)	2000 (457)	3200 (1380)	NR
$AUC_{(0inf)}$ (h*ng/mL)	3560 (845)	5210 (1700)	NR
$t_{1/2}$ (h)	3.60 (0.461)	3.58 (0.600)	NR
CL/F (L/h)	5.25 (1.14)	3.66 (1.08)	NR
Vdss/F (L)	26.9 (4.89)	18.5 (4.78)	NR

NR = Summary parameters not reported for Cohort 3.
Cohort 1 = IV: 2.5 mg/kg colistin base activity q12h x 2 doses, Aerosol: 75 mg colistin base activity q12h x 2 doses.
Cohort 2 = IV: 2.5 mg/kg colistin base activity q8h x 3 doses, Aerosol: 75 mg colistin base activity q8h x 3 doses.
Cohort 3 = IV: 3.3 mg/kg colistin base activity q8h x 3 doses, Aerosol: 75 mg colistin base activity q6h x 4 doses.
PK Parameters were based on the serial plasma collections taken after the administration of the final dose during the study period.
a. median (min-max).

Table S 6. Mean (SD) Plasma PK Parameters for CMS A in Period 1 by Cohort

Parameter	Cohort 1	Cohort 2	Cohort 3
C_{max} (ng/mL)	17400 (3220)	19700 (5530)	26700 (4630)
t_{max} (h)[a]	0.583 (0.500 – 0.667)	0.583 (0.583 – 1.00)	0.533 (0.500 – 0.667)
$AUC_{(0-t)}$ (h*ng/mL)	23100 (4180)	28600 (11900)	35900 (13000)
$AUC_{(0inf)}$ (h*ng/mL)	23800 (4390)	29400 (12000)	36800 (13200)
$t_{1/2}$ (h)	0.807 (0.163)	1.29 (0.558)	1.07 (0.625)
CL/F (L/h)	14.3 (2.81)	12.3 (3.91)	13.6 (5.62)
Vdss/F (L)	16.5 (4.20)	20.7 (6.52)	17.2 (6.10)

Cohort 1 = IV: 2.5 mg/kg colistin base activity q12h x 2 doses (total exposure: 5 mg/kg).
Cohort 2 = IV: 2.5 mg/kg colistin base activity q8h x 3 doses (total exposure: 7.5 mg/kg).
Cohort 3 = IV: 3.3 mg/kg colistin base activity q8h x 3 doses (total exposure: 10 mg/kg).
PK Parameters were based on the serial plasma collections taken after the administration of the final dose during the study period.
a. median (min-max).

Table S7. Mean (SD) Plasma PK Parameters for CMS A in Period 2 by Cohort

Parameter	Cohort 1	Cohort 2	Cohort 3
C_{max} (ng/mL)	662 (135)	716 (313)	1210 (792)
t_{max} (h)[a]	0.783 (0.350 – 1.92)	0.883 (0.367 – 0.967)	1.33 (0.800 – 1.80)
$AUC_{(0-t)}$ (h*ng/mL)	1370 (339)	1050 (471)	2260 (2240)
$AUC_{(0inf)}$ (h*ng/mL)	2350 (659)	3520 (3100)	5220 (-)
$t_{1/2}$ (h)	2.84 (0.965)	3.37 (4.39)	1.10 (-)
CL/F (L/h)	68.7 (18.9)	63.1 (31.2)	29.2 (-)
Vdss/F (L)	264 (31.1)	169 (82.2)	46.3 (-)

Cohort 1 = Aerosol: 75 mg colistin base activity q12h x 2 doses.
Cohort 2 = Aerosol: 75 mg colistin base activity q8h x 3 doses.
Cohort 3 = Aerosol: 75 mg colistin base activity q6h x 4 doses.
PK Parameters were based on the serial plasma collections taken after the administration of the final dose during the study period.
a. median (min-max).

Table S8. Mean (SD) Plasma PK Parameters for CMS A in Period 3 by Cohort

Parameter	Cohort 1	Cohort 2	Cohort 3
C_{max} (ng/mL)	14900 (4330)	15100 (5690)	NR
t_{max} (h)[a]	0.505 (0.333 – 0.618)	0.483 (0.267 – 0.700)	NR
$AUC_{(0-t)}$ (h*ng/mL)	21800 (3350)	27800 (14100)	NR
$AUC_{(0inf)}$ (h*ng/mL)	22700 (3690)	30600 (17200)	NR
$t_{1/2}$ (h)	1.07 (0.509)	1.18 (0.673)	NR
CL/F (L/h)	14.9 (2.96)	12.8 (4.95)	NR
Vdss/F (L)	22.6 (11.1)	18.8 (4.28)	NR

NR = Summary parameters not reported for Cohort 3.
Cohort 1 = IV: 2.5 mg/kg colistin base activity q12h x 2 doses, Aerosol: 75 mg colistin base activity q12h x 2 doses.
Cohort 2 = IV: 2.5 mg/kg colistin base activity q8h x 3 doses, Aerosol: 75 mg colistin base activity q8h x 3 doses.
Cohort 3 = IV: 3.3 mg/kg colistin base activity q8h x 3 doses, Aerosol: 75 mg colistin base activity q6h x 4 doses.
PK Parameters were based on the serial plasma collections taken after the administration of the final dose during the study period.
a. median (min-max).

Table S9. Mean (SD) Plasma PK Parameters for CMS B in Period 1 by Cohort

Parameter	Cohort 1	Cohort 2	Cohort 3
C_{max} (ng/mL)	2500 (472)	2610 (711)	3510 (841)
t_{max} (h)[a]	0.583 (0.500 – 0.667)	0.583 (0.583 – 1.00)	0.542 (0.500 – 0.667)
$AUC_{(0-t)}$ (h*ng/mL)	2870 (977)	2910 (1320)	3640 (1200)
$AUC_{(0inf)}$ (h*ng/mL)	3260 (1150)	3220 (1430)	4050 (1330)
$t_{1/2}$ (h)	1.04 (0.630)	0.682 (0.302)	0.857 (0.405)
CL/F (L/h)	13.3 (4.41)	13.5 (4.64)	13.5 (3.79)
Vdss/F (L)	17.1 (5.79)	11.8 (1.55)	15.6 (41.5)

Cohort 1 = IV: 2.5 mg/kg colistin base activity q12h x 2 doses (total exposure: 5 mg/kg).
Cohort 2 = IV: 2.5 mg/kg colistin base activity q8h x 3 doses (total exposure: 7.5 mg/kg).
Cohort 3 = IV: 3.3 mg/kg colistin base activity q8h x 3 doses (total exposure: 10 mg/kg).
PK Parameters were based on the serial plasma collections taken after the administration of the final dose during the study period.
a. median (min-max).

Table S10. Mean (SD) Plasma PK Parameters for CMS B in Period 3 by Cohort

Parameter	Cohort 1	Cohort 2	Cohort 3
C_{max} (ng/mL)	2110 (862)	1820 (878)	NR
t_{max} (h)[a]	0.505 (0.333 – 0.618)	0.492 (0.267 – 0.983)	NR
$AUC_{(0-t)}$ (h*ng/mL)	2690 (641)	2970 (2040)	NR
$AUC_{(0inf)}$ (h*ng/mL)	3130 (671)	3520 (2380)	NR
$t_{1/2}$ (h)	1.00 (0.413)	1.13 (0.836)	NR
CL/F (L/h)	13.0 (2.88)	13.7 (5.66)	NR
Vdss/F (L)	18.0 (5.61)	18.1 (4.45)	NR

NR = Summary parameters not reported for Cohort 3.
Cohort 1 = IV: 2.5 mg/kg colistin base activity q12h x 2 doses, Aerosol: 75 mg colistin base activity q12h x 2 doses.
Cohort 2 = IV: 2.5 mg/kg colistin base activity q8h x 3 doses, Aerosol: 75 mg colistin base activity q8h x 3 doses.
Cohort 3 = IV: 3.3 mg/kg colistin base activity q8h x 3 doses, Aerosol: 75 mg colistin base activity q6h x 4 doses.
PK Parameters were based on the serial plasma collections taken after the administration of the final dose during the study period.
a. median (min-max).

Supplementary Data S1. Diagnosis and Criteria for Inclusion and Exclusion

Potential subjects had to fulfilled all of the following inclusion criteria at baseline to be eligible for participation in the study:

1. Informed consent obtained and signed.
2. Aged between 18 and 45 years, inclusive.
3. Body mass index (BMI, weight in kg divided by the square of height in meters) between 18 kg/m^2 and 35.0 kg/m^2, inclusive.
4. Able to comply with protocol requirements for the entire duration of the study.
5. Healthy on the basis of a Screening medical evaluation (including physical examination, vital signs, blood biochemistry and hematology, urinalysis [UA], and history).

Potential subjects could not meet any of the following exclusion criteria to be eligible for participation in the study:

1. Heterosexually active females of child-bearing potential, defined as being physiologically capable of becoming pregnant, unless they agreed to use two of the following acceptable methods of contraception throughout their participation in the study and for at least 12 weeks after the final dose: (a) established use of oral, injected, or implanted hormonal contraception, (b) intrauterine device (IUD or Coil), (c) a female barrier method (diaphragm or cervical/vault cap), and/or (d) condom plus spermicidal cream/gel.
2. Heterosexually active males unless they agreed to use two concomitant acceptable methods of contraception throughout their participation in the study and for at least 12 weeks after receiving their final dose of study medication (examples included: vasectomy combined with latex condom with spermicide, latex condom with spermicide combined with a female partner who practices an acceptable method of contraception as indicated above).
3. History or current abuse of alcohol, barbiturates, amphetamines, tetrahydrocannibol, phencyclidine, cocaine, heroin, or other narcotics, as evidenced by a reported history or positive screen for these agents.
4. Any clinically significant (CS) (as deemed by the Principal Investigator [PI]) history of: asthma; cardiovascular, pulmonary, hepatic, renal, hematologic, gastrointestinal (including eating disorders), endocrine, metabolic, immunologic, dermatologic, neurologic (including a history of seizures, ataxia, or Myasthenia Gravis), psychological, or psychiatric disease; and/or a past or family history of porphyria.
5. Use of tobacco/nicotine within 3 months prior to Screening and for the entire duration of the study.
6. Treatment with another investigational drug 60 days prior to and/or during the study.
7. Co-enrollment in another study involving the intake of medication.
8. Immunocompromised status, including a positive human immunodeficiency virus-type 1 (HIV-1) or human immunodeficiency virus-type 2 (HIV-2) test by enzyme-linked immunosorbent assay (ELISA) at Screening.
9. Previously demonstrated CS allergy or hypersensitivity to colistimethate sodium or its excipients.
10. Donation of blood or significant blood loss within 56 days of study Enrollment or during study duration, or plasma donation within 28 days preceding study Enrollment.
11. Hepatitis B or C infection (confirmed by hepatitis B surface antigen [HBsAg], or hepatitis C virus antibody [HCV Ab], respectively) at Screening.
12. Laboratory abnormalities at Screening as outlined below:
    a. Serum creatinine ($\geq$1.1 × upper limit of normal [ULN])
    b. Hemoglobin (Hgb) (<11.0 g/dL or >17.5 g/dL)

   c. Platelet count (<125,000 or >450,000/mm^3)
   d. Absolute neutrophil count (ANC) (<1300/mm^3)
   e. Serum blood urea nitrogen (BUN) (≥1.2 × ULN)
   f. Aspartate aminotransferase (AST) (≥1.2 × ULN)
   g. Alanine aminotransferase (ALT) (≥1.2 × ULN)
   h. Proteinuria (spot urine) greater than trace and/or hematuria greater than trace

   Note: Subjects could undergo a repeat screening test of out-of-range analyte(s) at the discretion of the investigator to confirm a plausible alternative explanation that would be indicated in the source documentation. A repeat laboratory test could be used to satisfy eligibility requirements.

13. Intake of any of the following medications within 30 days prior to Screening and during the study: acyclovir, adefovir, aminoglycosides, amphotericin, cisplatin, cyclosporine, fluoroquinolones, foscarnet, ganciclovir, pamidronate, sirolimus, tacrolimus, and vancomycin, and/or any neuromuscular blockers.
14. Intake of non-steroidal anti-inflammatory drugs (NSAIDs) (ibuprofen, naproxen, etodolac) within 48 hours of dosing and any inhaled medication within 5 days of dosing. Additionally, subjects could have been excluded due to intake of medications not listed here at the discretion of the PIs.
15. Forced expiratory volume in 1 second (FEV1) < 80% predicted.
16. Prior evidence (symptoms within the past year) of vestibular problems or neuropathy.
17. Abnormal QT interval at Screening electrocardiogram (ECG) (Bazett correction > 450 milliseconds) or significant abnormities according to the cardiologist’s final reading.
18. A Grade 3 or 4 clinical or confirmed laboratory toxicity, which does not return to Grade 2 or lower.
19. Any condition that could have, in the opinion of the investigator, placed the subject at an unacceptable risk or injury, or rendered the subject unable to meet the requirements of the protocol.

Supplementary Data S2. Pharmacokinetic Sampling Schedule

Plasma for PK samples consisted of 3 mL of blood per sample. Post-dose samples 30 min to 4 hr had a ± 10 min window; post-dose samples > 4 hr to 8 hr had a ±15 min window; and samples > 8 hr had a ± 30 min window.

Cohorts 1 and 2

- Pre-dose (≤ 10 minutes)
- Immediately after the last dose in each dosing period (≤ 10 minutes), and 0.5, 1, 1.5, 2, 4, 8, 12, and 24 hr post-dose

Cohort 3

Dosing Period 1:

- Dose 1: within 15 minutes pre-dose, immediately post-dose, then 30 minutes, and 1 and 2 hours post-dose completion
- Dose 2: within 10 minutes pre-dose, immediately post-dose, then 30 minutes, and 1 hour post-dose completion
- Dose 3: within 10 minutes pre-dose, immediately post-dose, then 30 minutes, and 1, 2, 4, 6, 8, 12, 16, and 24 hours post-dose completion

Dosing Period 2:

- Dose 1: within 15 minutes pre-dose, then 30 minutes, and 1 and 2 hours post-dose completion
- Doses 2 and 3: within 10 minutes pre-dose, then 30 minutes, and 1 and 2 hours post-dose completion
- Dose 4: within 10 minutes pre-dose, then 30 minutes, and 1, 2, 4, 6, 7.5, and 24 hours post-dose completion

Dosing Period 3:

- IV Dose 1: within 15 minutes pre-dose, then immediately post-dose before starting the aerosol Dose 1
- Aerosol Dose 1: immediately pre-dose (see IV Dose 1), immediately post-dose, then 30 minutes, and 1.5 and 5 hours after completion of aerosol Dose 1
- Aerosol Dose 2: immediately post-dose and 30 minutes post-dose completion
- IV Dose 2: within 5 minutes pre-dose, immediately post-dose, then 30 minutes and1 hour post-dose completion
- Aerosol Dose 3: immediately post-dose, then 1, 1.5, and 2.5 hours post-dose completion
- IV Dose 3: within 5 minutes pre-dose, immediately post-dose, and 30 minutes post-dose completion
- Aerosol Dose 4: within 5 minutes pre-dose, then 30 minutes, and 1.5, 2, 3.5, 5.5, 7.5, 9.5, 12, and 24 hours post-dose completion

Supplementary Data S3. Serum Kidney Biomarker Time Points

Cohorts	Period 1	Period 2	Period 3
Cohorts 1 and 2	– Before dosing<br>– 24 hr after completion of all doses	– Before dosing<br>– 24 hr after completion of all doses	– Before dosing<br>– After completion of all doses<br>– Follow-up Visit
Cohort 3	– Dose 1: 0 to 2 hr pre-dose; then 30 min and 2 hr post-dose completion<br>– Dose 2: within 10 min pre-dose; then 1 and 5 hr post-dose completion<br>– Dose 3: within 10 min pre-dose; then 30 min and 2, 4, 8, 12, 16, and 24 hr post-dose completion	– Dose 1: 0 to 2 hr pre- dose; then 30 minutes and 2 and 5 hr post-dose completion<br>– Dose 2: none<br>– Dose 3: within 10 min pre-dose; then 30 min and 2 and 5 hr post-dose completion<br>- Dose 4: 1, 2, 4, 7.5, and 24 hr post-dose completion	– IV Dose 1: 0 to 2 hr pre-dose<br>– Aerosol Dose 1: immediately post- dose and 1.5 hr post-dose completion<br>– IV Dose 2: within 5 mins pre-dose; then 1 hr post-dose completion<br>– IV Dose 3: within 5 min pre-dose; then 30 min post-dose completion<br>– Aerosol Dose 4: within 5 min pre- dose; then 1, 5.5, 8.5, 13, and 24 hr post-dose completion<br>– Follow-up Visit
Cohort 4	– Before dosing<br>– 30 min after Dose 12<br>– 24 hours after completion of all 21 doses	– Before dosing<br>– 30 min after Dose 16<br>– 24 hr after completion of all 28 doses	– Before dosing<br>– 30 min after Dose 28<br>– 24 hr after completion of all 49 doses<br>– Follow-up Visit

hr = hour/hours, min = minute.

Supplementary Data S4. Urine Sample for Kidney Biomarkers

Cohorts	Period 1	Period 2	Period 3
Cohorts 1 and 2	– Before dosing<br>– 24 hr after completion of all doses	– Before dosing<br>– 24 hr after completion of all doses	– Before dosing<br>– 24 hr after completion of all doses<br>– Follow-up Visit
Cohort 3[a]	– Before dosing<br>- 0 to 2, 2 to 4, 4 to 8, 8 to 12, 12 to 24, and > 24 hr after the start of the first dose	– Before dosing<br>- 0 to 2, 2 to 4, 4 to 8, 8 to 12, 12 to 24, and > 24 hr after the start of the first dose	– Before dosing<br>- 0 to 2, 2 to 4, 4 to 8, 8 to 12, 12 to 24, and > 24 hr after the start of the first dose<br>– Follow-up Visit
Cohort 4	– Before dosing<br>– 30 minutes after Dose 12 and 24 hr after completion of all 21 doses	– Before dosing<br>– 30 minutes after Dose 16 and 24 hr after completion of all 28 doses	– Before dosing<br>– 30 minutes after Dose 28 and 24 hr after completion of all 49 doses<br>– Follow-up Visit

hr = hour/hours.

a. During each time period, a single 10-mL aliquot of urine was collected pre-dose and after start of first dose between 0 2, 2 4, 4-8, 8-12, 12-24 and >24 hours; the date and time for each sample was recorded.

Supplementary Data S5. Clinical Laboratory Evaluations

Hematology with Differential and Coagulation	CBC (WBC, Hgb, platelet count) with differential, PT, APTT
Serum Chemistry	creatinine, CPK, total bilirubin, BUN, AST, ALT, AP, glucose, sodium, potassium, chloride, bicarbonate, calcium, albumin, total protein
Urinalysis Dipstick	pH, specific gravity, protein, nitrite, blood, leukocyte esterase, microscopy (if indicated)
Additional Tests	HIV-1, -2 ELISA, HBsAg, HCV Ab, hCG (if applicable), urine drug screen (alcohol, cannabinoids, cocaine, amphetamines, opiates, benzodiazepines, barbiturates)
Kidney Biomarkers	serum cystatin C, NGAL, serum β microglobulin levels, urine microalbumin, urine creatine, urine protein

Supplementary Data S6. Schedule of Events – Cohorts 1 Through 4, All Dosing Periods

	Screening	Enrollment/ Re-admission	Cohort-specific Procedures				Discharge	Follow-up
**Procedure**	**≤ 28 days prior to first dose (1×/subject)**	**Before dosing in all 3 dosing periods (3×/subject)**	**Cohort 1**	**Cohort 2**	**Cohort 3**	**Cohort 4**	**24 hours after completion of the last dose in all 3 dosing periods (3×/subject)**	**2 weeks (±5 business days) after discharge in dosing Period 3 (1×/subject)**
Eligibility assessment	X	X						
Informed consent	X							
Assessment of AEs		X	X	X	X	X	X	X
Medical history	X	X						
Complete physical exam	X	X						X
Targeted physical exam			X	X	X	X	X	
Vital signs	X		X	X	X	X	X	
Hematology with differential & coagulation	X	X				X	X	X
Kidney biomarkers		X	X	X	X	X		X
Serum chemistry (after fasting)	X	X			X	X	X	X
Urinalysis dipstick	X	X				X	X	X
Urine alcohol/drug screen	X	X						
Urine hCG (females only)		X						X
Serum hCG (females only)	X							
Serum HIV-1, -2 ELISA, HBsAg, HCV	X							
Spirometry	X	X	X	X	X	X		X
12-Lead ECG	X						X	X
Administration of colistimethate sodium			X	X	X	X		
PK sampling			X	X	X	X		
Bronchoalveolar lavage (BAL)			X	X	X	X		
SARA Assessment Form	X	X	For each instance of ataxia one form at onset + one form prior to discharge					
Baseline Headache Assessment Form	X	(X)						
Infusion Headache Assessment Form							X (dosing Period 1 only)	

AE = adverse event, BAL = bronchoalveolar lavage, ECG = electrocardiography, ELISA = enzyme-linked immunosorbent assay, hCG = human chorionic gonadotrophin, HBsAg = hepatitis B surface antigen, HCV= hepatitis C virus, HIV = human immunodeficiency virus, PK = pharmacokinetic, UA = urinalysis, SARA = Scale for the Assessment and Rating of Ataxia
